# A case report of a severe form of cogan syndrome

**DOI:** 10.1016/j.amsu.2021.103036

**Published:** 2021-11-20

**Authors:** Abdoul Salam Youssoufou Souley, Moctar Issiakou, Oumarou Sambou Khidrou Fadhoullahi, Brah Souleymane, Samir Mainassara Chékaraou, Zeinabou Noura

**Affiliations:** a Ophthalmology Department of the Army Hospital, Niamey, Niger; b General Hospital of Reference of Niamey, Niger; c Military Hospital of Instruction Mohamed V, Morocco; d Faculty of Dentistry of Rabat, Morocco; e CHU Ibn Rochd Rabat, Morocco

**Keywords:** The cogan syndrome, Goniosynechia, Optic atrophy

## Abstract

**Introduction:**

the Cogan syndrome is a very rare, systemic disease that affects young adults. Very few cases are described in the literature. We report the case of a patient with a severe form of Cogan syndrome.

**Case presentation:**

This is a young patient who presented with a painful left red eye and bilateral visual impairment evolving for 5 years with ENT signs such as right hypoacusis and vertigo. Clinical examination in this patient found bilateral hypertensive panuveitis, vertigo of peripheral origin and hypoacusis on the right. The patient is currently on corticosteroid therapy with stabilization of the lesions.

**Discussion:**

This pathology is characterized by ocular and audio-vestibular involvement with sometimes other visceral manifestations. The etiopathogeny is not well known, the evolution is marked by the functional prognosis (visual and auditory) and the vital prognosis (aortic insufficiency). The treatment is essentially based on corticotherapy and the treatment of complications.

**Conclusion:**

It is a very rare condition that should be considered when there are suggestive signs, because the evolution is severe without appropriate care. This underlines the importance of early management and the need for optimal follow-up to avoid the occurrence of complications that are disabling or even fatal.

## Highlights

• The complications of the Cogan syndrome.
• Goniosynechia over 360*.
• Retinal ischemia.
• Optic atrophy.

## 1 Introduction

The Cogan syndrome is defined by the association of a non-syphilitic interstitial keratitis and a cochleovestibular involvement, with various visceral manifestations which can thus realize the picture of a systemic disease. Cogan syndrome is a very rare systemic disorder that affects young adults without gender predilection [1]. Very few cases are described in the literature. We report the case of a patient with a severe form with ocular and cochleovestibular involvement, it is the first case diagnosed in Niger. This work has been reported in line with the SCARE 2020 criteria [2].

## 2 Case presentation

This is a 40 year old patient with no personal or family medical history, who consulted for a painful red left eye and a decrease in bilateral visual acuity. The onset was 5 years ago with the appearance of a painful left eye and a decrease in visual acuity with an evolution by relapse and remission for which the patient was put on anti-inflammatory treatment with the notion of poor therapeutic compliance.

In fact, the appearance of the ocular signs was contemporary with that of the ENT signs made of a right hypoacusis and vertigo repeatedly (with a frequency of 3/year), which initially responded to corticosteroid therapy, then became more and more resistant. In 2018 a sero-mucosal otitis was objectified indicating a drainage by transtympanic aerator and the patient was put under corticosteroid therapy for 6 weeks with a good initial improvement and a recurrence of hypoacusis after one year of inertia, and worsening of vertigo in 2021.

### 2.1 The clinical examination reveals

**Fig. 1:**
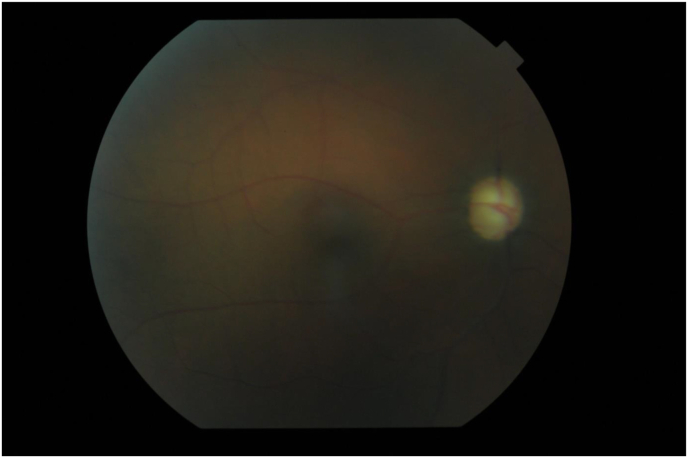
The right eye: papillary pallor evoking an optic atrophy.

In the right eye a visual acuity of 8/10, a normal cornea without dystrophy; a fine tyndall in the anterior chamber and an intraocular pressure of 40 mm Hg. On gonioscopy, 360° goniosynechia were observed. At the fundus, there was a vitreous tyndall and optic atrophy (Fig. 1).

**Fig. 2:**
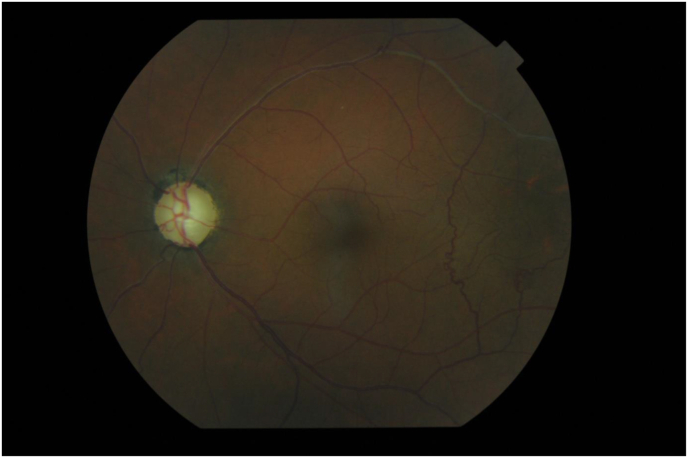
Disinhabited blood vessels temporally superior to the fundus.

The visual acuity of the left eye was 1/10. On examination, we noted a tyndall in the anterior chamber, an intraocular pressure of 38 mm Hg; more numerous goniosynechia over 360°; a vitreous tyndall, blood vessels in the superior temporal region of the fundus and an optic atrophy (Fig. 2).

On otoscopic examination, a pocket of antero-inferior retraction was found in the right ear; the eardrum had a purplish appearance.

The Rinne test is positive in the left ear and negative in the right ear.

The weber test is lateralized to the left.

The rest of the clinical examination came back without any particularity.

The search for other etiologies came back negative, notably infectious, immunological and inflammatory causes. The serologies of infectious diseases; the immunological assessments as well as the assessments of diseases such as Harada's disease, sarcoidosis, all came back negative.

The tonal audiometry objectives a Perception Deafness with a loss of 63.75 dB on the left and a Mixed Deafness with a loss of 87.5 dB on the right.

On tympanogram: the curve is flattened on the right and normal on the left.

**Fig. 3:**
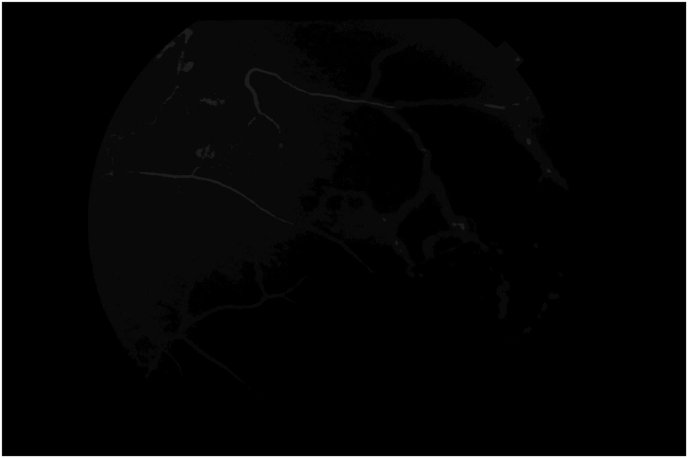
The fluorescence angiography shows peripheral retinal ischemia in the left eye with retinal vasculitis.

The retinal angiography shows retinal ischemia in the superior temporal region with diffusion of the contrast product. At the level of the papilla, there is a papillary pallor evoking an optic atrophy (Fig. 3).

**Fig. 4:**
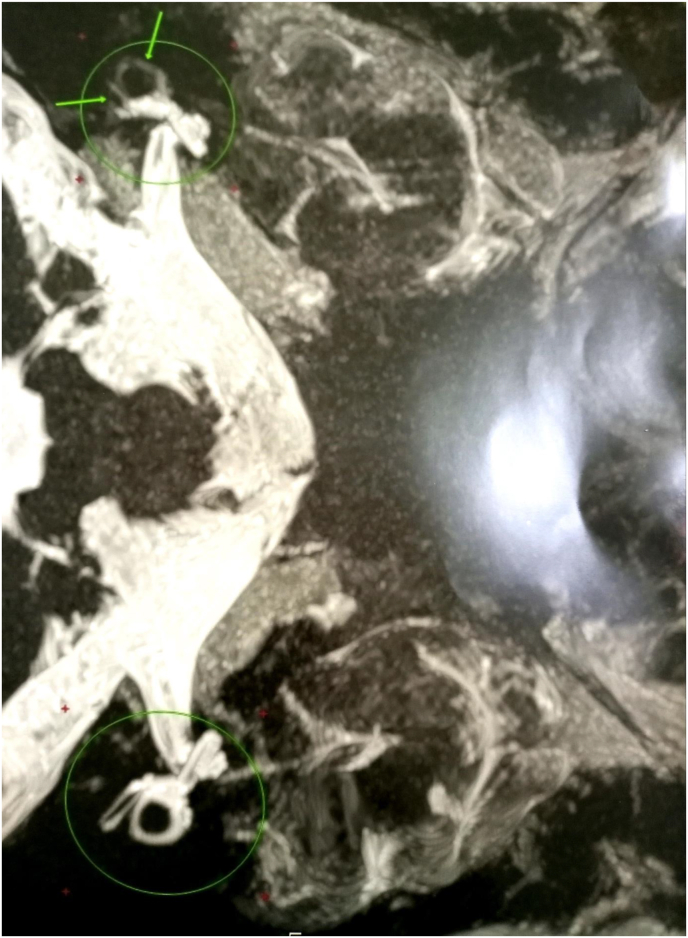
Focal obliteration of the semicircular canals with a spiky appearance of their lumens. Appearance in favor of an ossifying labyrinthitis.

The cerebral MRI reveals a focal obliteration of the semicircular canals with a spiky aspect of their lumens. Appearance in favor of an ossifying labyrinthitis (Fig. 4).

The prognosis for this patient was severe, with irreversible lesions, notably optic atrophy and deafness.

The patient was put on high dose corticosteroid therapy at a rate of 1 m/kg for 2 weeks and then progressive degression, anti glaucoma therapy and anti vertigo drugs with a better control of the disease.

## 3 Discussion

Cogan syndrome is an autoimmune disease defined by a non-syphilitic interstitial keratitis associated with audio-vestibular involvement [[3], [4]], affecting another organ in 2 cases out of 3 and presenting a true systemic disease, reminiscent of vasculitis in one case out of three. The diagnosis is based on the suggestive clinical association and the elimination of other causes of stromal keratitis (e.g., syphilis, Lyme disease, Epstein-Barr virus infection) [5].

The incidence of Cogan's syndrome is not known; in France, a retrospective study noted 30 cases diagnosed in 15 years. Cogan's syndrome mainly affects young adults; the onset is one out of two between 20 and 30 years of age and in 8/10 of the cases between 10 and 40 years of age; the extremes of age are 3 months and a half and 81 years with an average age of 30 years; the majority of the cases described concern Caucasian subjects, with no predominance of sex (52% of men for 48% of women) [1].

The pathophysiology is not yet clearly established, but an immune reaction directed against the constituents of the cornea is suspected. Ocular manifestations simulate retinal vasculitis [6].

Clinically we have ocular signs (interstitial keratitis, scleritis, conjunctivitis, anterior uveitis, lacrimation and photophobia and episcleritis), audio-vestibular (vertigo, hypoacusis, nystagmus). There may be other manifestations: general (fever, weight loss); cardiac (especially aortic insufficiency); arterial (murmur, abolition of pulse, abdominal pain, intermittent claudication, ischemic necrosis of the extremities); musculoskeletal (polyarthralgia, myositis), neurological (Pyramidal syndrome, Cerebellar syndrome …) , cutaneous-mucosal signs (vascular purpura, nodules or ulcerations), digestive (diarrhea, rectorrhagia, moelena, mesenteric arteritis) [1].

Biological examinations are not specific and may reveal an inflammatory syndrome in the acute phase [6], and sometimes immunological abnormalities, there is no specific immunological test that could reveal this pathology.

In front of the association of an ocular and audio vestibular attack, we can evoke as diagnostic hypothesis: Congenital syphilis (which is an exclusion criterion for cogan syndrome); Vogt-Koyanagi-Harada syndrome (which associates audio-vestibular involvement with uveitis, alopecia, poliosis, and vitiligo); Susac syndrome (which manifests itself by attacks associating in a variable way a decrease in visual acuity, deafness and central neurological disorders); finally, systemic diseases such as PAN, sarcoidosis, atrophying polychonritis, rheumatoid arthritis, systemic lupus erythematosus, Horton's disease [1].

The etiopathogeny of this affection remains unknown, however one notes in certain cases an infection or an exposure has toxic a few days before the beginning of the first symptoms, without a direct link being established.

The evolution of the disease is characterized by ocular and/or vestibular flare-ups, between which an apparently complete remission can be observed. The prognosis is determined by the risk of permanent deafness, ocular damage and by cardiovascular complications, in particular aortic insufficiency, which can lead to the patient's death.

Treatment is based on topical and systemic corticosteroid therapy. Corticosteroids have an effect on eye signs but not on auditory symptoms [7].as was the case with our patient.

Immunosuppressants are proposed in case of cortico-resistance or cortico-dependence.

## 4 Conclusion

Cogan syndrome is a systemic disease with ocular and vestibular predominance. Ocular involvement when detected early can be well treated and prevent the occurrence of serious complications as in our patient's case. This underlines the need for early and multidisciplinary management and regular follow-up. Treatment is based on corticosteroid therapy and treatment of complications.

## Ethics approval

Not related as this is a case report.

## Sources of funding

Not available.

## Authors contributions

**YOUSSOUFOU SOULEY ABDOUL SALAM**: Corresponding author.

**SOULEYMANE BRAH**: study concept.

**OUMAROU FADHOULLAHI**: data analysis.

**SAMIR CHEKAROUA MAINASSARA**: wrote and reviewed the final paper.

**MOCTAR ISSIAKOU**: contributed to the diagnosis.

**ZEINABOU NOURA**: data collection.

## Trial registry number

This is a case report. No human participants were involved.

## Consent

Written informed consent has been obtained from the patient and parents for publication of this case report and accompanying images. A written copy of the consent is available for review by the editor-in-chief of this journal.

## Guarantor

Dr. Youssoufou Souley Abdoul Salam is the guarantor of this manuscript.

## Sourcing and peer review

Not commissioned, externally peer reviewed.

## Declaration of competing interest

The authors declare that there are no conflicts of interest.

## References

[1] Vinceneux P., Pouchot J., Kahn M.F., Peltier A.P., Meyer O., Piette J.C. (1991).

[2] Agha R.A., Franchi T., Sohrabi C., Mathew G., pour le groupe S.C.A.R.E. (2020). Ligne directrice SCARE 2020 : mise à jour des lignes directrices du rapport sur les cas chirurgicaux de consensus (SCARE). Int. J. Surg..

[3] Cogan D.G. (1963). Non-syphilitic interstitial keratitis with vestibulo-auditory symptoms: a case with fatal aortitis. Trans. Am. Ophthalmol. Soc..

[4] Cundiff J., Kansal S., Kumar A., Goldstein D.A., Tessler H.H. (2006). Cogan's syndrome: a cause of progressive hearing deafness. Am. J. Otolaryngol..

[5] Vollertsen R.S., McDonald T.J., Younge B.R., Banks P.M., Stanson A.W., Ilstrup D.M. (1986). Cogan's syndrome: 18 cases and a review of the literature. Mayo Clin. Proc..

[6] Gauthier A.S., Delbosc B. (2012). Kératites interstitielles. EMC - Ophtalmol..

[7] Norton E.W., Cogan D.G. (1959). Syndrome of nonsyphilitic interstitial keratitis and vestibuloauditory symptoms; a long-term follow-up. *AMA* Arch. Ophthalmol..

